# M-CSF Signals through the MAPK/ERK Pathway via Sp1 to Induce VEGF Production and Induces Angiogenesis *In Vivo*

**DOI:** 10.1371/journal.pone.0003405

**Published:** 2008-10-14

**Authors:** Jennifer M. Curry, Tim D. Eubank, Ryan D. Roberts, Yijie Wang, Nabendu Pore, Amit Maity, Clay B. Marsh

**Affiliations:** 1 The Dorothy M. Davis Heart and Lung Research Institute, The Ohio State University, Columbus, Ohio, United States of America; 2 The Integrated Biomedical Science Graduate Program, College of Medicine and Public Health, The Ohio State University, Columbus, Ohio, United States of America; 3 Department of Internal Medicine, The Ohio State University, Columbus, Ohio, United States of America; 4 Department of Radiation Oncology, University of Pennsylvania School of Medicine, Philadelphia, Pennsylvania, United States of America; Oklahoma Medical Research Foundation, United States of America

## Abstract

**Background:**

M-CSF recruits mononuclear phagocytes which regulate processes such as angiogenesis and metastases in tumors. VEGF is a potent activator of angiogenesis as it promotes endothelial cell proliferation and new blood vessel formation. Previously, we reported that *in vitro* M-CSF induces the expression of biologically-active VEGF from human monocytes.

**Methodology and Results:**

In this study, we demonstrate the molecular mechanism of M-CSF-induced VEGF production. Using a construct containing the VEGF promoter linked to a luciferase reporter, we found that a mutation reducing HIF binding to the VEGF promoter had no significant effect on luciferase production induced by M-CSF stimulation. Further analysis revealed that M-CSF induced VEGF through the MAPK/ERK signaling pathway via the transcription factor, Sp1. Thus, inhibition of either ERK or Sp1 suppressed M-CSF-induced VEGF at the mRNA and protein level. M-CSF also induced the nuclear localization of Sp1, which was blocked by ERK inhibition. Finally, mutating the Sp1 binding sites within the VEGF promoter or inhibiting ERK decreased VEGF promoter activity in M-CSF-treated human monocytes. To evaluate the biological significance of M-CSF induced VEGF production, we used an *in vivo* angiogenesis model to illustrate the ability of M-CSF to recruit mononuclear phagocytes, increase VEGF levels, and enhance angiogenesis. Importantly, the addition of a neutralizing VEGF antibody abolished M-CSF-induced blood vessel formation.

**Conclusion:**

These data delineate an ERK- and Sp1-dependent mechanism of M-CSF induced VEGF production and demonstrate for the first time the ability of M-CSF to induce angiogenesis via VEGF *in vivo*.

## Introduction

Macrophage-Colony Stimulating Factor (M-CSF) acts on mononuclear phagocytes, stimulating proliferation, driving differentiation of monocytes to macrophages, and protecting these cells against apoptosis [1]–[3]. Many cell types, including fibroblasts [4], monocytes [5], endothelial cells [6] and tumor cells [7] secrete M-CSF. M-CSF recruits mononuclear phagocytes to sites of injury or inflammation by itself [8] or through a paracrine loop involving monocyte chemoattractant protein-1 (CCL2/MCP-1) [9]. Upon binding M-CSF, the M-CSF receptor dimerizes and elicits intrinsic tyrosine kinase activity triggering signaling pathways that induce cell survival. Using human monocytes, we previously demonstrated that M-CSF activates the phosphatidylinositol 3-kinase/Akt (PI3K/Akt) pathway, which induces cell survival by suppressing caspase-9 and caspase-3 activation [1]. Also, M-CSF activates the pro-survival mitogen-activated protein kinase (MAPK) pathway, including ERK, p38, and JNK [10]–[13].

VEGF is a potent activator of angiogenesis as it promotes endothelial cell proliferation and new blood vessel formation [14]. During hypoxic conditions, the Hypoxia-Inducible Factor (HIF) proteins regulate VEGF production. At low oxygen levels, the HIFs are stabilized and bind promoter sites known as hypoxia regulatory elements (HREs), driving the transcription of over 20 hypoxia-inducible genes [15]. During normoxia, VEGF can be expressed from cells by hypoxia-independent mechanisms still involving the HIF apparatus. For example, ERK can phosphorylate and activate HIF-1α thereby increasing VEGF expression [16]. Alternatively, VEGF can be expressed in a HIF-independent fashion. In cancer cells lines, Akt activation through Sp1 increases VEGF expression and augments angiogenesis *in vivo* [17]. Also, activation of Raf-1 (an upstream regulator of the MEK/ERK pathway) can induce VEGF expression in hamster fibroblast cells [18].

M-CSF, mononuclear phagocytes, and VEGF are linked to poor prognosis in patients with solid tumors. For example, M-CSF is elevated in breast cancer patients with advanced disease and distant metastases compared to patients with localized tumors [19], [20]. In addition, a higher peripheral blood monocyte count is related to tumor progression and is an independent risk factor for recurrence of hepatocellular carcinoma after resection [21]. Finally, the importance of VEGF in tumor progression is well-established. In breast and lung cancer patients, VEGF expression negatively correlates with relapse-free and overall survival [22], [23]. Additionally, a monoclonal antibody against VEGF, Bevacizumab, inhibits tumor growth by blocking angiogenesis and is approved for use with chemotherapy to treat metastatic colon cancer [24] and metastatic non-small cell lung cancer [25]. Because of the critical effects of angiogenesis and VEGF on tumor growth and metastases and the clear negative prognostic role of M-CSF and VEGF in human tumors, we sought to define the biochemical mechanisms underlying M-CSF-induced VEGF production. This approach extends our previous observations that M-CSF directly induces functional VEGF production from human monocytes [26].

Here, we decipher the molecular mechanism of M-CSF-induced VEGF production. Further, we illustrate the *in vivo* ability of M-CSF to induce VEGF production and cause subsequent angiogenesis.

## Material and Methods

### Purification of peripheral blood monocytes

Single donor human monocytes were isolated from leukocyte source packs obtained from the American Red Cross (Columbus, OH) as described previously [27].

### Inhibitor studies

Human monocytes (10×10^6^ cells/condition) were left untreated, treated with DMSO or methanol, the MEK/ERK inhibitor, U0126 (10 µM), the JNK inhibitor, SP600125 (10 µM), the p38 MAP kinase inhibitor, SB203580 (10 µM), the PI3K/Akt inhibitor, LY294002 (1 µM or 10 µM), the mTOR inhibitor, Rapamycin (0.1, 1, or 10 µM), or mithramycin (8, 40, and 200 nM) for 30 minutes (Calbiochem, San Diego, CA). Afterwards, the cells were stimulated with rhM-CSF (100 ng/ml) for 48 hours and the cell-free supernatants were frozen until assayed for VEGF by ELISA (R&D Systems, Minneapolis, MN).

### Western blot analysis

Protein was harvested using Cell Lysis Buffer from Cell Signaling. Whole cells lysates were standardized using the Bio-Rad Protein Assay Kit. Western blots were performed using NuPAGE® 10% Bis-Tris gels, NuPAGE® MOPS SDS Running Buffer, and NuPAGE® Transfer Buffer (Invitrogen) and subsequently transferred to nitrocellulose paper and probed with the following antibodies: phosphorylated p44/p42 MAPK (Thr202/Tyr204) (#9101L, Cell Signaling) and equal loading was observed by blotting for total ERK2 (sc-154, Santa Cruz).

### Sp1 Transcription Factor Binding Assay

Human monocytes were either left non-stimulated or stimulated with rhM-CSF (100 ng/ml) for 30 minutes. Nuclear lysates were isolated from the cells using the CelLytic™ NuCLEAR™ Extraction Kit (Sigma). All samples were normalized for total protein using the Bio-Rad Protein Assay Kit. The amount of Sp1 in the nuclear lysates was analyzed using the NoShift™ Transcription Factor Assay Kit and NoShift™ Sp1 reagents as described by the manufacturer (Novagen).

### Confocal microscopy

Human monocytes were left non-stimulated or stimulated with rhM-CSF (100 ng/ml) for 6 or 24 hours. Cells were fixed then permeablized with 1% Triton X-100 and subsequently blocked with 5% goat serum in PBS, incubated overnight with 1 µg/ml of either a primary antibody to human Sp1 (Upstate) or IgG control antibody (Santa Cruz). The cells were incubated with a 1∶5000 dilution of Alexa 594-conjugated secondary antibody (Molecular Probes). The slides were then viewed and imaged using the Zeiss LSM 510 confocal microscope. Images were quantified using Image J software. Cells were differentiated from debris by using the phase contrast photos and only those cells displaying monocyte morphology, horseshoe-shaped nuclei, were included in the analysis.

### VEGF promoter analysis

All luciferase reporter constructs containing both wild type and mutant sequences based on the VEGF promoter were supplied by Dr. Amit Maity (University of Pennsylvania). 1 µg of each construct was transfected into freshly-isolated human monocytes using the Amaxa Nucleofector Transfection System as per the manufacturer's protocol (Amaxa). All cells were incubated in 100 ng/ml rhM-CSF for 16 hours, lysed using Promega Lysis Buffer on ice, and centrifuged to remove cell debris. 20 µl of each cell lysate was mixed with 90 µl luciferase substrate and read on the luminometer in triplicate.

### VEGF mRNA analysis

Monocyte total RNA was collected using the Absolutely RNA™ RT-PCR Miniprep Kit Stratagene® (La Jolla, CA) for total RNA purification as previously described [26]. cDNA was synthesized using the Gibco BRL SuperScript First-Strand Synthesis System for RT-PCR Kit. Real-time polymerase chain reaction was performed using the human VEGF forward primer (5′-AGGCCAGCACATAGGAGAGATG-3′), and reverse primer (5′-CAAGGCCCACAGGGATTTTC-3′). The primers for VEGF mRNA were designed based on the human VEGF-A sequence obtained from PubMed (Genbank accession no. NM003376). Reaction conditions were as follows: 10 µl SYBR® Green PCR Master Mix (Applied Biosystems), 0.4 µl each forward and reverse VEGF primers (10 µM) or primers targeting GAPDH (control), 8.2 µl RNase-free water, and 1.0 µl cDNA (0.5 µg) for a total reaction of 20 µl. The real-time PCR was completed on the ABI PRISM Sequence Detector 7700 (PerkinElmer) using Sequence Detector software. Reaction conditions were as follows: 50°C for 2 min, 95°C for 10 min, and 40 cycles of 95°C for 15 s and 60°C for 1 min. Quantification was performed as previously described [28].

### In vivo Matrigel™ plug assay

C57BL/6 female mice purchased from Jackson Laboratories (Bar Harbor, ME) were anesthetized with isoflurane and subcutaneously injected with 0.5 ml growth factor-reduced Matrigel™ matrix (Discovery Labware, Bedford, MA) supplemented with either PBS, rmVEGF (10 ng/ml), rmM-CSF (100 ng/ml), rmM-CSF (100 ng/ml)+anti-VEGF neutralizing antibody (5 µg/ml), or rmM-CSF (100 ng/ml)+isotype IgG antibody (5 µg/ml). All components added to the unpolymerized Matrigel™ were allowed to incubate at 4°C for at least 12 hours prior to injection. After 10 days, the mice were sacrificed and the Matrigel™ plugs removed, formalin-fixed, paraffin-embedded and sectioned.

### Immunohistochemistry

Mononuclear phagocyte recruitment within the plugs was identified using an anti-F4/80 antibody (Serotec, MCAP497). VEGF was detected within the plugs by staining with a rabbit anti-VEGF antibody or the isotype IgG antibody (LabVision, RM-9128-R7). Endothelial cell infiltration into the plugs was determined by an anti-von Willebrand factor (vWf) antibody (Abcam, ab6994). Blood vessels within each plug are defined as complete circles of vWf(+)-stained cells. All staining was performed at the Pathology Core at The Ohio State University.

### Statistical analyses

Statistics were determined by The Ohio State University Medical Center for Biostatistics. For IHC of F4/80(+) cells and VEGF and the Sp1 binding assay, an independent samples Student's T-Test was used to assess differences between groups. An ANOVA with Tukey's post-hoc test was performed to determine differences between groups in the blood vessel counts, ELISA studies, confocal microscopy data and the VEGF promoter constructs data. For the inhibitor studies and the luciferase assays, difference in fold change over treatment was tested using a random-intercept mixed model. Fold change was log transformed for variance stabilization and a single overall *p*-value that tests if there is a difference between treatments was generated. All analyses were run on Stata 10.0, Stata Corporation, College Station, Texas. Groups were considered significantly different at p<0.05.

## Results

### M-CSF-induced VEGF production from monocytes occurs through a HIF-independent mechanism

**Figure 1:**
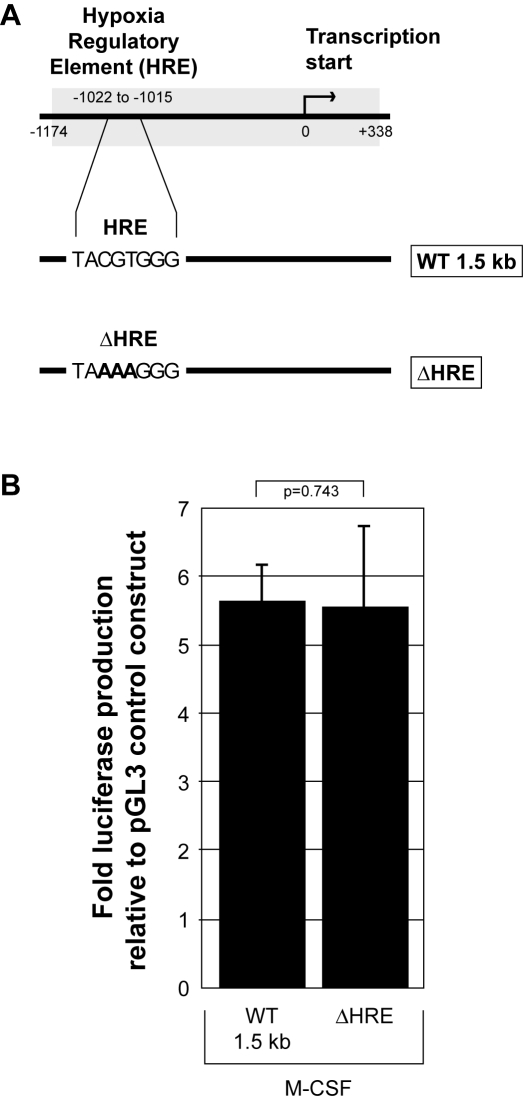
M-CSF induced VEGF production occurs through a HIF-independent mechanism. A) The 1.5 kb wild type VEGF promoter *(WT1.5 kb)* and the same promoter sequence containing a non-functional mutation of the hypoxia regulatory element (HRE) *(ΔHRE)* were inserted into the pGL3-basic vector to create constructs that produce luciferase upon VEGF promoter activation. Nucleotides changed from the original wild type sequence are designated in bold. Human monocytes were transfected with either the control empty construct (pGL3-Basic), or pGL3 containing 1 µg of each of the described VEGF promoter constructs above. B) Transfected monocytes containing *(WT1.5 kb)* or *(ΔHRE)* were allowed to adhere for 1 hour in RPMI/5% FBS followed by the addition of fresh media containing rhM-CSF (100 ng/ml) for 16 hours. The adherent cells were lysed and assayed for luciferase production using a luminometer. This data represents the mean+/−SEM of six individual blood donors. All data is represented as the fold change in luciferase over the pGL3 control construct.

We demonstrated M-CSF induces the production and release of biologically active VEGF from human monocytes [26]. Here, we elucidate the biochemical mechanism of this phenomenon by first investigating the role of the hypoxia-inducible factors (HIFs). During hypoxic stress, HIFs are the master regulators of VEGF and function by binding to the hypoxia regulatory element (HRE) sequence within the VEGF promoter. We utilized both wild type and mutant VEGF promoter constructs driving luciferase production. We transfected human monocytes with either the pGL3-basic empty vector, the same pGL3 construct containing a 1.5 kb portion of the wild type VEGF promoter, or a VEGF promoter construct containing a mutated HRE to prohibit HIF protein binding (**Figure 1A**). This assay measures luciferase production as a function of VEGF promoter activation. All cells were transfected using the Amaxa Nucleofector, stimulated with M-CSF, and subsequent luciferase production was measured at 16 hours and represented as fold-change over the luciferase production from the pGL3 empty vector. When transfecting the eGFP control construct into human monocytes, we observed an efficiency of 55.3% as analyzed by flow cytometry (data not shown). Cells expressing the VEGF wild type construct displayed a 4.5-fold increase in luciferase production compared to the pGL3-basic empty vector control (p = 0.020). Mutation in the HRE did not affect the luciferase production (p = 0.743) suggesting M-CSF-induced VEGF promoter activation is HIF-independent (**Figure 1B**).

### ERK plays a regulatory role in M-CSF-induced VEGF production

**Figure 2:**
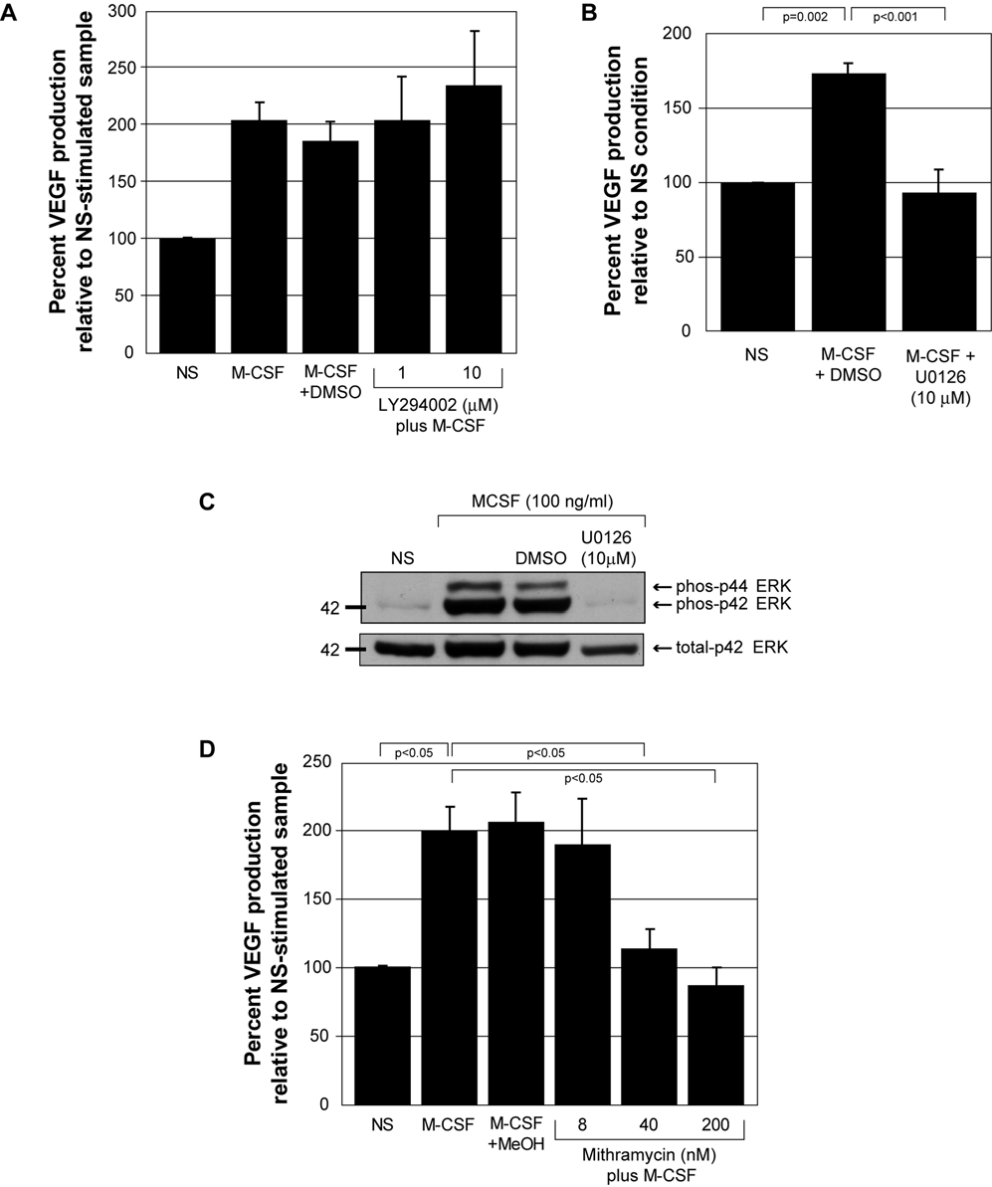
M-CSF-induced VEGF production from human monocytes is blocked by ERK inhibition and mithramycin. A) Monocytes were pre-incubated with DMSO (vehicle control), 1 µM or 10 µM the PI3 kinase inhibitor (LY294002) for 30 minutes. The monocytes were either left non-stimulated *(NS)*, stimulated with rhM-CSF (100 ng/ml) *(M-CSF)*, with rhM-CSF (100 ng/ml) plus DMSO *(M-CSF+DMSO)*, or rhM-CSF (100 ng/ml) plus 1 or 10 µM LY294002 *(1)* and *(10)* for 48 hours. Cell-free supernatants were evaluated for VEGF by ELISA. These data represent the mean±SEM from four independent donors. B) Monocytes were pre-incubated with DMSO (vehicle control) or 10 µM of the inhibitor of ERK activity (U0126) for 30 minutes. The monocytes were either left non-stimulated *(NS)*, stimulated with rhM-CSF (100 ng/ml) plus DMSO *(M-CSF+DMSO)* or with rhM-CSF (100 ng/ml) plus U0126 *(M-CSF+U0126 (10 µM))* for 48 hours. Cell-free supernatants were evaluated for VEGF by ELISA. These data represent the mean±SEM from six independent donors. C) Freshly-isolated human monocytes were plated overnight in 5% FBS and M-CSF (20 ng/ml). The next day the cells were starved for 2 hours in minimal media. For the last 30 minutes, DMSO (vehicle control) or 10 µM U0126 was added to the appropriate samples. The cells were left non-stimulated *(NS)*, stimulated with rhM-CSF (100 ng/ml) *(M-CSF+DMSO)* or *(M-CSF+U0126)* for 10 minutes. Cell lysates were probed for phospho-ERK MAP Kinase (Thr202) and total ERK MAP Kinase by western blot analysis. This data is representative of two independent monocyte donors. D) Monocytes were pre-incubated with methanol (vehicle control) or 8, 40, or 200 nM of the Sp1 transcription factor binding inhibitor, mithramycin, for 45 minutes. The cells were either left non-stimulated *(NS)* or stimulated with rhM-CSF (100 ng/ml) *(M-CSF)*, *(M-CSF+MeOH)*, *(M-CSF plus 8 nM, 40 nM, or 200 nM mithramycin)* for 48 hours. Cell-free supernatants were evaluated for VEGF by ELISA. These data represent the mean±SEM from ten independent blood donors.

M-CSF activates both the PI3 kinase/Akt pathway [1] and MAP kinase pathway, including ERK [29], p38 [30], and JNK [31]. Both over-expression of Akt and activation of ERK can drive VEGF production in cancer cell lines or in hamster fibroblasts, respectively [32], [33]. Therefore, we investigated the roles of Akt and the MAP kinases in M-CSF-induced VEGF production from primary human monocytes. We treated peripheral blood monocytes with pharmacological inhibitors targeting PI3 kinase, the upstream activator of Akt (LY294002), mTOR, a signaling molecule downstream of Akt (Rapamycin), JNK (SP600125), p38 (SB203580), or ERK (U0126) and stimulated these cells with M-CSF. We analyzed VEGF levels in the supernatants by ELISA. Our data suggested that the PI3-K/Akt pathway did not regulate M-CSF-induced VEGF production as neither LY294002 nor Rapamycin affected VEGF production (p = 0.898 for 1 µM and p = 0.334 for 10 µM LY294002) (**Figure 2A**) and (p = 1.0 for 1 µM and p = 0.640 for 10 µM Rapamycin) (data not shown). Importantly, the inhibition of ERK activity by U0126 reduced VEGF production from M-CSF-stimulated monocytes to that produced from non-stimulated cells (p<0.001) (**Figure 2B**). Western blot analysis confirmed that M-CSF induced ERK1/2 phosphorylation, which was inhibited by U0126 (**Figure 2C**). Our data also indicated that M-CSF-induced VEGF was not regulated by either p38 or JNK as inhibitors targeting these pathways did not affect M-CSF-induced VEGF production (p = 0.44 and p = 0.62, respectively) (data not shown). Cell viability in response to all inhibitors was analyzed by Trypan blue exclusion and was not adversely affected (data not shown).

### Mithramycin inhibits VEGF production

Sp1 and AP-2 are transcription factors phosphorylated and activated by ERK [34] and depicted as regulators of VEGF [35]. To investigate the regulatory role of Sp1 or AP-2 in M-CSF-induced VEGF production, we cultured human monocytes and pre-treated with methanol (vehicle control) or mithramycin, an inhibitor that intercalates into GC-rich regions of DNA and inhibits the binding of transcription factors like Sp1 and AP-2. Monocytes were left untreated or treated with M-CSF for 48 hours and VEGF levels in the supernatants were analyzed by ELISA. Mithramycin (40 nM and 200 nM) significantly inhibited M-CSF-induced VEGF production compared to vehicle-treated cells (p<0.05, respectively) (**Figure 2D**). Cell viability as assessed by Trypan Blue exclusion was not affected in cells treated with mithramycin up to 200 nM (data not shown).

To verify whether the inhibitory effect of mithramycin was due to inhibition of Sp1 binding, we attempted to use siRNA against Sp1. Stealth™ RNAi targeting Sp1 was transfected into primary human monocytes using Lipofectamine and assayed for a reduction in total Sp1 protein by Western blot. We found that protein levels of Sp1 were not depleted from these cells until 48 hours after transfection. This created a challenge in VEGF determination as cultured, primary human monocytes differentiate into macrophages which become less responsive to M-CSF and therefore make it difficult to measure VEGF production from these cells. Additionally, transfection of monocytes with Lipofectamine alone induced the production of VEGF that masked induction of VEGF by M-CSF (data not shown). As a result, we attempted to transfect the siRNA using the Amaxa Nucleofector. Using this method, transfection alone induced the production of the soluble form of VEGFR-1, which sequesters VEGF from antigenic detection by ELISA. For these reasons, we chose to perform multiple techniques described below to verify the role of Sp1 in M-CSF-induced VEGF production from these cells.

### Sp1 is a downstream activator in M-CSF-induced VEGF production

**Figure 3:**
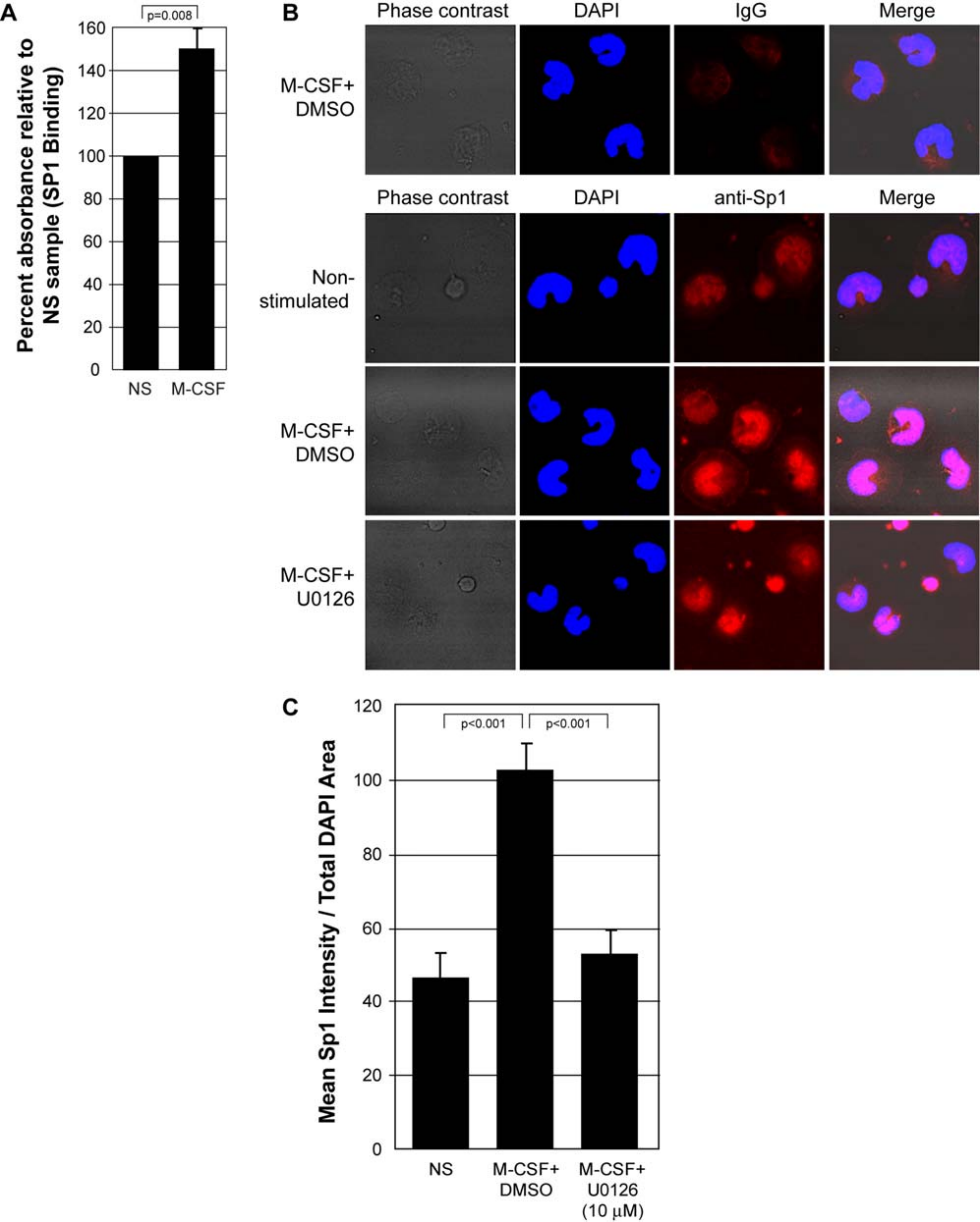
M-CSF induces Sp1 nuclear localization in an ERK-dependent manner. A) Human monocytes were left non-stimulated *(NS)* or stimulated with rhM-CSF (100 ng/ml) *(M-CSF)* for 30 minutes. Nuclear lysates were isolated and normalized for total protein. Sp1 that translocated into the nucleus in response to M-CSF was analyzed using a biotinylated Sp1 DNA sequence bound to a streptavidin-coated plate, a polyclonal rabbit anti-Sp1 primary antibody, a HRP-conjugated goat anti-rabbit IgG secondary antibody, and TMB substrate. The absorbance was read at 450 nm to reflect Sp1 within the nucleus. These data represent the mean±SEM from four independent blood donors. B) Human monocytes were starved for 6 hours, inhibited for 30 minutes with U0126 (10 µM) or DMSO (vehicle control) and left non-stimulated *(Non-stimulated)* or treated with rhM-CSF for 6 hours *(M-CSF+DMSO)* and *(M-CSF+U0126)*. The cells were fixed, permeablized, and stained with a normal IgG control antibody *(top row)* or a primary antibody targeting Sp1 followed by subsequent staining with Alexa 594-conjugated secondary antibody, targeting Sp1 *(red)*, and with DAPI stain designating the nucleus *(blue)*. Images were captured using the Zeiss LSM 510 confocal microscope. These pictures are representative of four individual monocyte donors. C) Quantification of Sp1 localization to the nucleus of monocytes (horseshoe-shaped nuclei) using Image J software. This data represents mean+/−SEM of cells from four individual trials.

The activation of Sp1 during M-CSF stimulation has not been previously reported. To confirm that M-CSF induces Sp1 activation, we performed an Sp1 binding assay to measure levels of Sp1 translocation to the nucleus in monocytes stimulated with M-CSF for 30 minutes. Our results indicate that M-CSF increased the amount of Sp1 that migrated to the nucleus of human monocytes (p = 0.008) (**Figure 3A**).

To investigate the role of Erk in Sp1 nuclear translocation, we analyzed localization of Sp1 in human monocytes by confocal microscopy. Monocytes were pre-incubated with DMSO (vehicle control) or U0126 and left untreated or treated with M-CSF for 6 or 24 hours. The cells were fixed and stained using a fluorescently-conjugated anti-Sp1 antibody and DAPI to stain the nucleus (**Figure 3B**). Image J software analysis confirmed that M-CSF enhanced Sp1 nuclear localization compared to untreated monocytes (p<0.001). Furthermore, the ERK inhibitor, U0126, blocked Sp1 nuclear translocation compared to cells treated with M-CSF plus the vehicle control (DMSO) (p<0.001) (**Figure 3C**).

### M-CSF up-regulates VEGF mRNA transcription in an ERK- and Sp1-dependent manner

**Figure 4:**
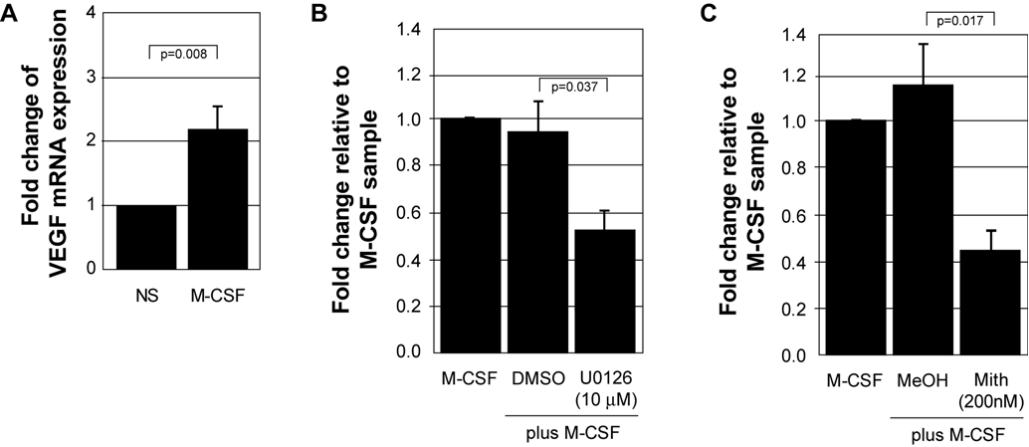
M-CSF regulates VEGF mRNA transcription through ERK and Sp1. A) Freshly isolated human monocytes were cultured overnight in 5% FBS and subsequently starved for 2 hours in minimal media. The cells were stimulated with 100 ng/ml rhM-CSF for 6 hours followed by isolation of RNA. cDNA was synthesized from total cellular RNA, standardized, and subjected to SYBR Green real-time PCR using primers specific for VEGF-A. The data represents the mean+/−SEM of six individual donors. B) Freshly isolated human monocytes were cultured overnight in 5% FBS and subsequently starved for 2 hrs followed by pre-treatment with either DMSO (vehicle control) or U0126 (10 µM) in minimal media for 30 minutes. The cells were then stimulated with 100 ng/ml rhM-CSF *(M-CSF)*, *(M-CSF+DMSO)*, *(M-CSF+U0126 10 µM)* and analyzed as stated in A. The data represents the mean+/−SEM of three individual donors. C) Freshly isolated human monocytes were cultured overnight in 5% FBS and subsequently starved for 2 hours followed by pre-treatment with either methanol (vehicle control) or mithramycin (200 nM) in minimal media. The cells were then stimulated with 100 ng/ml rhM-CSF *(M-CSF)*, *(M-CSF+MeOH)*, *(M-CSF+Mith (200 nM))* and analyzed as in A. The data represents the mean+/−SEM of four individual donors.

Next, we examined the role of Erk and Sp1 in the transcriptional regulation of VEGF mRNA. We isolated total RNA from monocytes left untreated or treated with M-CSF. Using Real-Time PCR, we found a significant increase in VEGF mRNA at 6 hours of M-CSF stimulation compared to the untreated sample (p = 0.008) (**Figure 4A**). Next, we isolated RNA from cells pre-incubated with U1026, mithramycin, or vehicle controls and treated with M-CSF. Real-time PCR analysis revealed that inhibiting the activity of ERK (U0126) (**Figure 4B**) or Sp1 (mithramycin) (**Figure 4C**) significantly decreased VEGF mRNA transcription (p = 0.037 and p = 0.017, respectively). These data further supports our hypothesis that M-CSF-induced VEGF production is transcriptionally regulated and is dependent on both ERK and Sp1 activity.

### ERK and Sp1 are essential for M-CSF to functionally regulate the VEGF promoter

**Figure 5:**
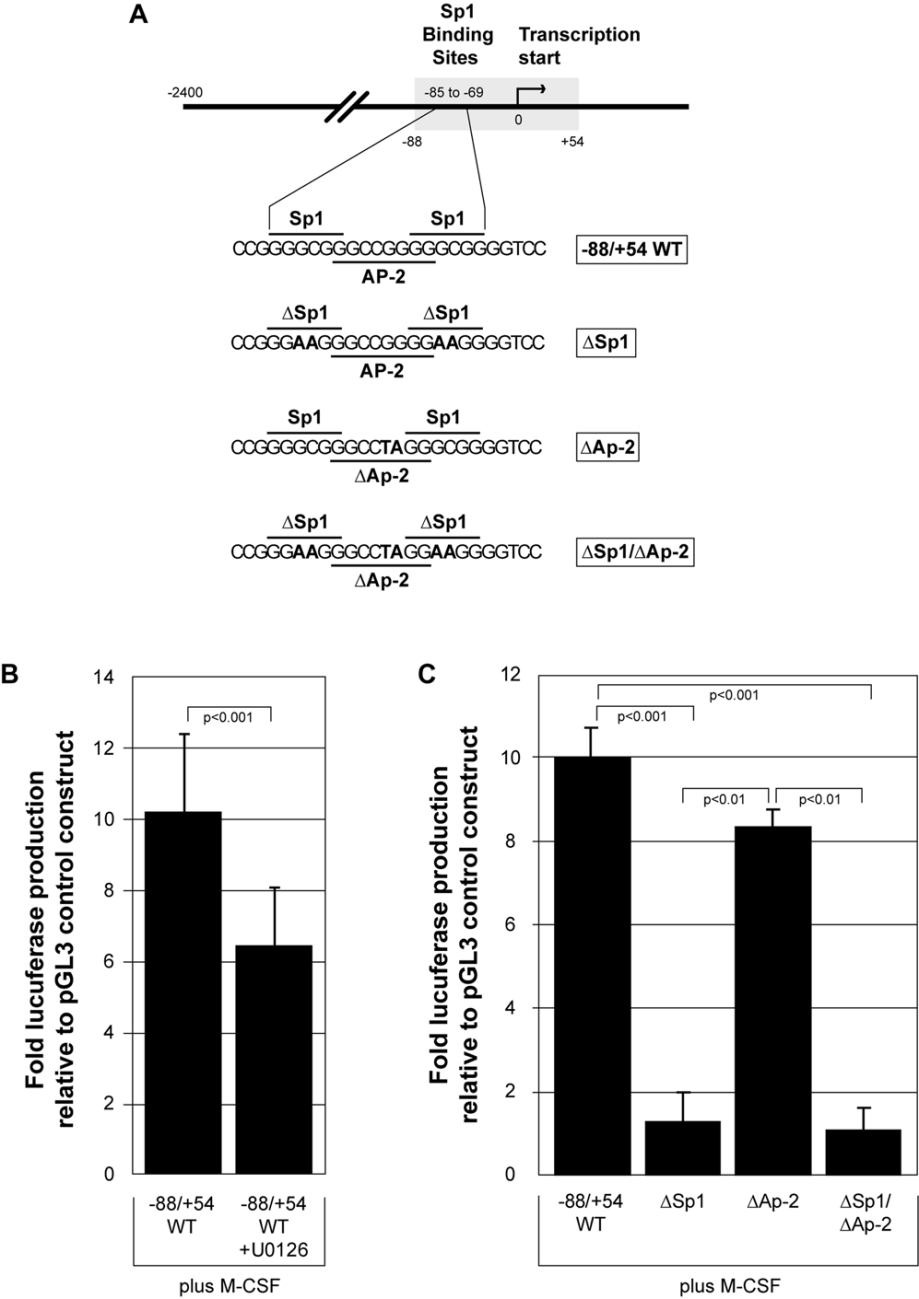
ERK and Sp1 are necessary for M-CSF induced functional activation of the VEGF promoter. A) The wild type VEGF promoter (region −88 to +54 relative to the transcription start site (0)) *(−88/+54 WT)* and the same promoter sequence containing mutations of Sp1 (*ΔSp1*), AP-2 (*ΔAP-2*), and a Sp1/AP-2 double mutant (*ΔSp1/ΔAP-2*) were inserted into the pGL3-basic vector to drive luciferase production upon VEGF promoter activation. Nucleotides changed in the mutated sequences are designated in bold. B) Transfected monocytes containing the pGL3 control vector or *(−88/+54 WT)* were allowed to adhere for 1 hour in RPMI/5% FBS followed by the addition of 10 µM U0126 *(−88/+54 WT+U0126)* for 30 minutes. The cells were washed and fresh media containing rhM-CSF (100 ng/ml) was added for 16 hours, when the cells were lysed and assayed for luciferase production using a luminometer. This data represents the mean+/−SD of six individual blood donors. C) Transfected monocytes containing *(−88/+54 WT)*, *(ΔSp1)*, *(ΔAP-2)*, or *(ΔSp1/ΔAP-2)* were allowed to adhere for 1 hour in RPMI/5% FBS followed by the addition of fresh media containing rhM-CSF (100 ng/ml). After 16 hours, adherent cells were lysed and assayed for luciferase production using a luminometer. This data represents the mean+/−SD of three individual blood donors.

To investigate the functional role of ERK and Sp1, we transfected primary human monocytes with wild type or mutant VEGF promoter constructs that drive luciferase production when activated. The VEGF wild type promoter region (+54 to −88); the identical region of the VEGF promoter with the two Sp1 regions (−85 to −80 and −74 to −69) mutated to inactivity by replacing GC to AA in both instances resulting in no Sp1 binding; an AP-2 mutant (−80 to −73) by changing the GG at −75 and −76 to TA; and an Sp1/AP-2 double mutant were spliced into the pGL3-basic vector to create the promoter constructs (**Figure 5A**). To interrogate the role of ERK in the functional activity of the VEGF promoter, we transfected monocytes with the VEGF wild type construct and inhibited ERK activity with U0126 treatment. After 16 hours of M-CSF stimulation, cells transfected with the VEGF wild type construct produced 10-fold more luciferase than the pGL3 control vector. Inhibition of ERK activation by U0126 caused a statistically significant reduction in luciferase production from M-CSF-stimulated cells compared to the cells challenged with M-CSF plus vehicle control (p<0.001) (**Figure 5B**).

Next, the VEGF mutant constructs were utilized to investigate the role of AP-2 in this system and to confirm the biological activity of Sp1 in response to M-CSF. Again, M-CSF induced a significant 10-fold increase in luciferase production from monocytes transfected with the VEGF wild type construct compared to cells transfected with the control pGL3 empty vector (p<0.001). Monocytes transfected with the Sp1 mutant or the Sp1/AP-2 double mutant produced levels of luciferase similar to cells transfected with the empty vector and significantly less than the cells transfected with the VEGF wild type construct (p<0.001 for both) (**Figure 5C**). Furthermore, the role of AP-2 in this system was minimal, as there was not a statistically significant reduction in luciferase produced by cells transfected with the AP-2 mutant construct compared to the wild type construct (p = 0.105). These data solidify our hypothesis that M-CSF induces VEGF production through the MAPK/ERK pathway via Sp1.

### M-CSF promotes mononuclear phagocyte recruitment, induces VEGF production, and enhances angiogenesis *in vivo*

**Figure 6:**
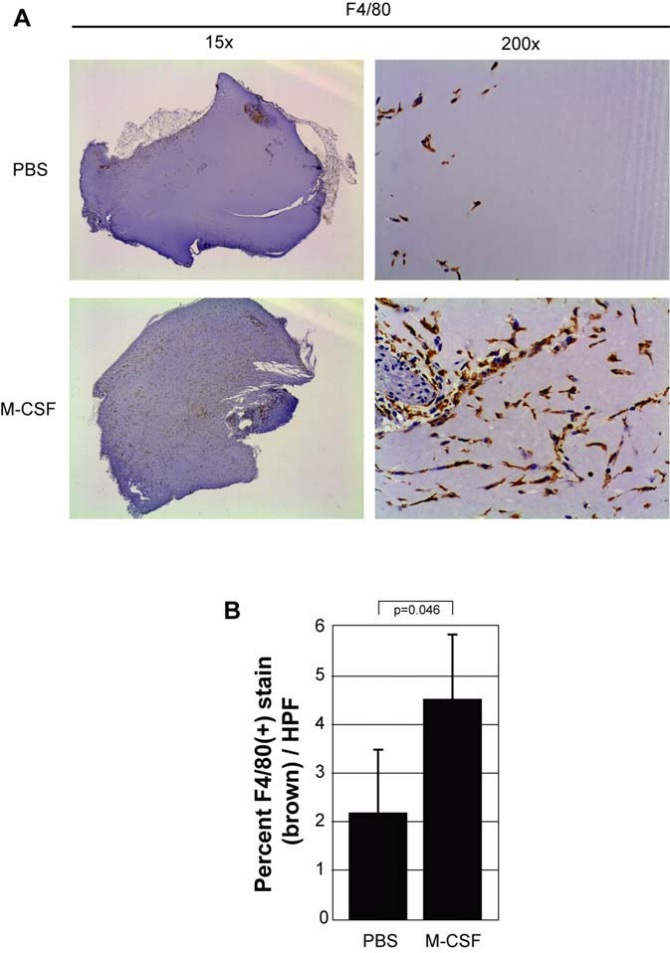
M-CSF induces mononuclear phagocyte recruitment *in vivo*. A) Matrigel™ was resuspended with PBS *(PBS)* or rmM-CSF (100 ng/ml) *(M-CSF)* at 4°C overnight and then injected subcutaneously into mice. Matrigel™ was left *in situ* for 10 days and then harvested. The plugs were stained for mononuclear phagocytes using an anti-mouse F4/80 antibody. Brown staining represents F4/80(+) cells within the plug. Pictures shown were taken using a dissecting microscope (1.5× objective lens) *(left)* to display overall mononuclear phagocyte influx between conditions as well as an inverted microscope (20× objective lens) *(right)* to show detailed staining of these cells. B) The percent of F4/80(+) cells (brown stain) per plug was evaluated from 15 digital images captured randomly in a blinded manner. Adobe Photoshop histogram pixel analysis of the brown stain was used for quantification. This data represents three plugs per group.

**Figure 7:**
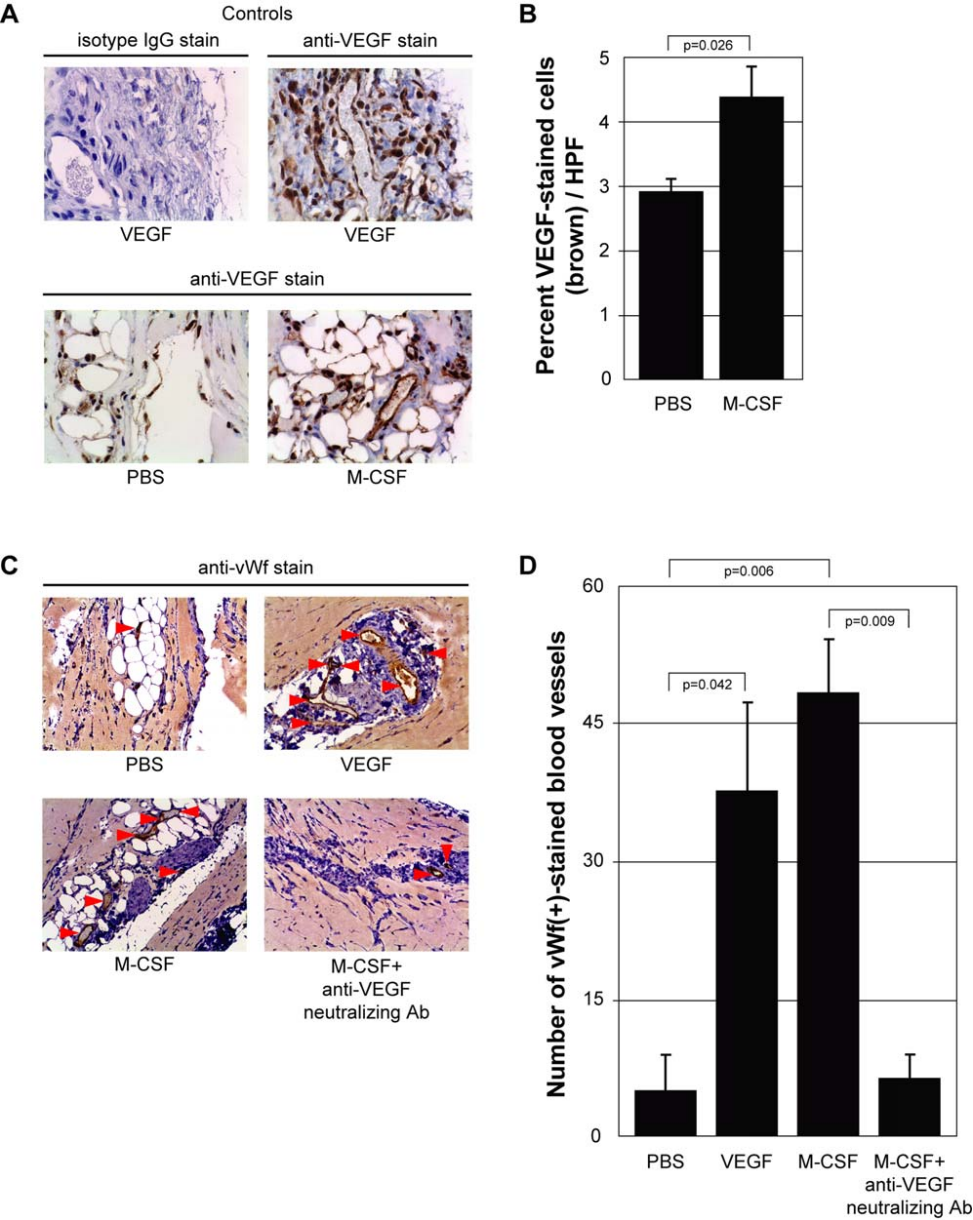
M-CSF induces VEGF and regulates angiogenesis *in vivo*. A) Matrigel™ was resuspended with PBS *(PBS)*, rmVEGF (10 ng/ml) *(VEGF)* or rmM-CSF (100 ng/ml) *(M-CSF)*. The slides were stained with either an anti-mouse VEGF antibody or its isotype IgG. Brown staining represents VEGF within the plug. Pictures were taken using a 20× objective lens. B) The amount of brown stain (VEGF) within the plugs was determined by taking 15 digital images randomly using a 20× objective lens in a blinded manner. Quantification of VEGF within each plug was determined by Adobe Photoshop histogram pixel analysis of the brown stain. C) Matrigel™ was resuspended with PBS *(PBS)*, rmVEGF (10 ng/ml) *(VEGF)*, rmM-CSF (100 ng/ml) *(M-CSF)*, or rmM-CSF (100 ng/ml)+anti-VEGF neutralizing antibody (5 µg/ml) *(M-CSF+anti-VEGF neutralizing Ab)*. The plugs were stained for endothelial cells using an anti-mouse von Willebrand factor (vWf) antibody. Brown staining represents vWf(+) cells within the plugs. Pictures were taken using a 20× objective lens. D) Quantification of blood vessels within each plug was determined by counting the total number of vWf(+)-stained endothelial cells that formed a complete circle (red arrows) throughout each entire plug using a 20× objective lens in a blinded manner. This data represents three plugs per group.

With the molecular mechanism of M-CSF-induced VEGF production established, we next investigated the *in vivo* role of VEGF induced by M-CSF. We performed an *in vivo* angiogenesis assay by mixing PBS, VEGF or M-CSF with Matrigel™ and subcutaneously injected the solution into the flanks of C57Bl/6 female mice. After 10 days, the plugs were removed and fixed for analysis by immunohistochemistry. First, we confirmed the ability of M-CSF to recruit mononuclear phagocytes by staining the PBS- and M-CSF-containing plugs with an antibody specific for F4/80, a marker of mononuclear phagocytes (**Figure 6A**). Significantly more F4/80(+) cells were present in the M-CSF-treated plugs compared to those plugs treated with PBS (p = 0.046) (**Figure 6B**). Next, we stained plugs containing PBS, VEGF (positive control), or M-CSF with an antibody for murine VEGF to investigate if the presence of M-CSF in the Matrigel plug augmented VEGF levels. Qualitatively, more VEGF was present in the M-CSF plugs compared to the PBS plugs. Additionally, the control stains demonstrate the specificity of the VEGF antibody as staining with IgG isotype antibody produced no positive areas (**Figure 7A**). Quantitatively, M-CSF significantly elevated the amount of VEGF (brown stain) in the plugs compared to those plugs with PBS (p = 0.026) (**Figure 7B**).

To investigate the angiogenic potential of VEGF produced by M-CSF within these plugs, we mixed PBS, VEGF, M-CSF, M-CSF plus an anti-VEGF neutralizing antibody, or M-CSF plus an isotype IgG antibody into Matrigel™ and injected into the flanks of mice. We analyzed blood vessel formation in the plugs by staining for von Willebrand factor (vWf), a glycoprotein present in blood plasma and produced by endothelial cells, and counted the number of vWf-positive cells lining blood vessel lumens (completely-lined circles) throughout the plug (**Figure 7C**). We found significant increases in blood vessels in plugs containing either VEGF or M-CSF compared to plugs containing PBS (p = 0.042 and p = 0.006, respectively). Furthermore, the number of blood vessels in plugs containing M-CSF plus a VEGF neutralizing antibody were significantly less than M-CSF-containing plugs and no different than PBS-containing plugs (p = 0.009 and p = 0.970, respectively) (**Figure 7D**). We observed no difference in blood vessel presence between plugs containing M-CSF and plugs containing M-CSF plus an isotype antibody (data not shown). These data solidify M-CSF-induced recruitment of mononuclear phagocytes and production of VEGF as an angiogenesis-inducing phenomenon.

## Discussion

We previously reported that human monocytes produce biologically-active VEGF in response to M-CSF stimulation [26]. This observation has been subsequently confirmed by Varney et al [36]. Here, we first investigated the molecular mechanisms involved in M-CSF-induced VEGF production from human monocytes. We provide data to support the hypothesis that M-CSF-induced VEGF production from human monocytes is mediated by ERK activation leading to the activation of Sp1. We found activated Sp1 translocated to the nucleus, binds to and induces VEGF promoter activation and results in VEGF production. Additionally, we found no role of the hypoxia inducible factors (HIFs) in this mechanism. Furthermore, for the first time, we demonstrate the angiogenic activity of M-CSF *in vivo* and illustrate that VEGF is the key molecule causing M-CSF-induced angiogenesis.

M-CSF can activate multiple cell signaling pathways that lead to VEGF production. For example, in murine cardiomyocytes, M-CSF functions through Akt to produce VEGF in response to ischemic injury, improving cardiac function [37]. Also, M-CSF activates the MAP kinases which play roles in VEGF production as ERK activation enhances VEGF promoter activity and p38 and JNK stabilize VEGF mRNA [34]. Using pharmacological inhibitors of PI3 kinase and mTOR, a downstream molecule in the Akt pathway, we found no role for the PI3 kinase/Akt pathway in M-CSF-induced VEGF production. Also, inhibition of p38 or JNK did not affect M-CSF induced VEGF production in human monocytes. However, it is possible that p38 and JNK can functionally compensate for each other as they have been shown to have the same mechanism of increasing VEGF levels by stabilizing VEGF mRNA [38].

The VEGF promoter contains binding sites for the transcription factors Sp1, AP-2 and the HIFs. Interestingly, ERK can induce the direct phosphorylation of both HIF-1α and Sp1 [16], [39]. The HIFs are known as the master regulators of VEGF and are responsible for the production of VEGF in response to hypoxia. However, HIFs can also induce VEGF production in non-hypoxic environments in response to cytokines and growth factors [40]. Varney et al demonstrated enhanced levels of HIF-1α after 72 hours of M-CSF stimulation and showed M-CSF-induced binding to the HRE by EMSA, suggesting a HIF-dependent mechanism [36]. However, when we mutated the HRE within the VEGF promoter, we did not observe any reduction in the levels of VEGF promoter activity in response to M-CSF. M-CSF may affect the stability of the HIFs and induce their binding to the HRE, but we did not observe a functional role for HIF binding in M-CSF-induced VEGF promoter activity.

The transcription factor Sp1 is activated by ERK and induces VEGF production in other cells. Milanini et al demonstrated a cooperative effect of AP-2 and Sp1 to activate the VEGF promoter. They transfected a fibroblast cell line with mutated VEGF promoter constructs and found that only the VEGF promoter construct with mutations in both the Sp1 and AP-2 binding sites displayed reduced promoter activation [18]. By performing similar promoter assay experiments, we found that a mutation in the Sp1 binding site alone was sufficient to reduce M-CSF-induced VEGF promoter activity to basal levels. Mutation of the AP-2 binding site within the VEGF promoter construct did reduce luciferase levels about 20%, however, this reduction was not statistically significant or comparable to the luciferase production observed with the Sp1 mutant. Therefore, we do not rule out a possible cooperative effect of Sp1 and AP-2 for VEGF promoter activation, but in our model of M-CSF stimulation of human monocytes, we found Sp1 to be the main transcription factor responsible for VEGF production.

Finally, we demonstrate the ability of M-CSF to induce angiogenesis *in vivo*. Using the Matrigel plug angiogenesis assay, we showed that M-CSF recruited mononuclear phagocytes, increased VEGF protein levels, and increased the number of blood vessels formed within the Matrigel plug. The recruitment of F480-positive cells was expected as M-CSF is a known chemoattractant for monocytes/macrophages, alone or through induction of monocyte chemoattractant protein (CCL2/MCP-1) by monocytes [10], [41]. Our observation of increased VEGF production and angiogenesis within the M-CSF-containing plugs confirms the ability of M-CSF to induce angiogenesis *in vivo*. In addition to inducing VEGF production from monocytes, M-CSF is also known to stimulate the production of other pro-angiogenic factors, such as IL-8 [38] and MMP-9 [43]. To decipher the angiogenic molecule responsible for the blood vessel formation in the M-CSF-containing plugs, we added a neutralizing antibody to VEGF to the M-CSF-containing plugs. The neutralization of VEGF within these plugs significantly reduced the angiogenesis implicating VEGF as a necessary molecule in M-CSF-induced angiogenesis.

In summary, we demonstrate the biochemical details of M-CSF-induced VEGF production. We propose a pathway involving M-CSF-induced ERK activation followed by ERK-induced phosphorylation of the transcription factor Sp1, which then translocates to the nucleus and binds to its specific sequence within the VEGF promoter to drive transcription of VEGF mRNA. Previous data on VEGF regulation has been obtained using cell lines; we demonstrate our phenomenon in primary human monocytes, enhancing the relevance of this observation. This manuscript describes the pro-angiogenic potential of M-CSF as it pertains to mononuclear phagocyte recruitment, VEGF production, and angiogenesis *in vivo*. The abundance of clinical data from cancer patients correlating high levels of M-CSF, mononuclear phagocytes, and VEGF with poor prognosis highlights the importance of increasing our understanding of this paradigm. We believe studies linking these three components may cultivate understanding and aid in the genesis of novel therapies for numerous cancers.
